# The host generalist phytopathogenic fungus *Sclerotinia sclerotiorum* differentially expresses multiple metabolic enzymes on two different plant hosts

**DOI:** 10.1038/s41598-019-56396-w

**Published:** 2019-12-27

**Authors:** Jefferson Allan, Roshan Regmi, Matthew Denton-Giles, Lars G. Kamphuis, Mark C. Derbyshire

**Affiliations:** Centre for Crop and Disease Management (CCDM), Curtin University, Kent Street, Perth, 6102 WA Australia

**Keywords:** Gene expression, Fungi

## Abstract

*Sclerotinia sclerotiorum* is a necrotrophic fungal pathogen that infects upwards of 400 plant species, including several economically important crops. The molecular processes that underpin broad host range necrotrophy are not fully understood. This study used RNA sequencing to assess whether *S. sclerotiorum* genes are differentially expressed in response to infection of the two different host crops canola (*Brassica napus*) and lupin (*Lupinus angustifolius*). A total of 10,864 of the 11,130 genes in the *S. sclerotiorum* genome were expressed. Of these, 628 were upregulated *in planta* relative to *in vitro* on at least one host, suggesting involvement in the broader infection process. Among these genes were predicted carbohydrate-active enzymes (CAZYmes) and secondary metabolites. A considerably smaller group of 53 genes were differentially expressed between the two plant hosts. Of these host-specific genes, only six were either CAZymes, secondary metabolites or putative effectors. The remaining genes represented a diverse range of functional categories, including several associated with the metabolism and efflux of xenobiotic compounds, such as cytochrome P450s, metal-beta-lactamases, tannases and major facilitator superfamily transporters. These results suggest that *S. sclerotiorum* may regulate the expression of detoxification-related genes in response to phytotoxins produced by the different host species. To date, this is the first comparative whole transcriptome analysis of *S. sclerotiorum* during infection of different hosts.

## Introduction

*Sclerotinia sclerotiorum* is a fungal pathogen of more than 400 plant species^1^. Because *S. sclerotiorum* feeds predominantly on dead tissue, it is generally described as a necrotroph. The hosts of *S. sclerotiorum* include a variety of economically important broadacre crop species, such as *Brassica napus* (canola), *Lupinus angustifolius* (lupin), *Glycine max* (soybean) and *Pisum sativum* (field pea)^1^. In Australia, *S. sclerotiorum* is best known as a pathogen of canola and costs the industry an estimated $10.1 million per year on average^2^.

Necrotrophic phytopathogens are thought to use a variety of pathogenicity factors to facilitate infection of their plant hosts. A variety of carbohydrate-active enzymes (CAZymes) are secreted by necrotrophs such as *S. sclerotiorum*, including xylanases, pectinases and cellulases. CAZymes that degrade plant cell walls are known more specifically as cell wall-degrading enzymes (CWDEs), and may facilitate the penetration of plant tissues by fungal appressoria^3^. In *S. sclerotiorum*, a suite of candidate CAZyme-encoding genes have been predicted and some have been characterised^4–6^. These include a variety of enzymes associated with the degradation of cell wall components such as cellulose, hemicellulose and pectins^4^. Many necrotrophs are also known to secrete broad-spectrum phytotoxic secondary metabolites to induce host tissue necrosis. Prominent examples include botrydial and botcinic acid, which are secreted by the broad host range necrotroph *Botrytis cinerea* during infection^7^. Infection assays have demonstrated that *B. cinerea* strains without the ability to produce these pathogenicity factors are substantially less virulent, suggesting that they form an important component of the infection process in *B. cinerea*^7^.

Fungal necrotrophs also have means of mitigating and manipulating host defence responses. Phytotoxic secondary metabolites produced by plant hosts during infection, such as phytoalexins, are metabolised by *S. sclerotiorum* and other necrotrophs to limit their effects^8–10^. Alternatively, plant-derived phytotoxins may be exported from fungal cells by transporter-based efflux^11,12^. However, fungal strategies for managing the plant immune response may be considerably more sophisticated than detoxification. For example, the infection process of *S. sclerotiorum* is thought to exhibit an early biotrophic phase, during which the pathogen secretes oxalic acid (OA) as a “compatibility factor” to subdue host defences^13,14^. Due to the presence of an early biotrophic phase, it has been suggested that *S. sclerotiorum* may be better described as a hemibiotroph than a necrotroph. This shift in paradigm, from the prototypical necrotroph infection strategy to one with an early biotrophic phase, is aligned with a broader concept that has emerged of a full continuum of trophic strategies among phytopathogens, in which necrotrophs may have more in common with biotrophs than once thought^14,15^.

Host-generalist necrotrophs such as *S. sclerotiorum* and *B. cinerea* differ substantially from other specialist necrotrophs such as *Alternaria spp*. and *Leptosphaeria maculans* in their ability to infect a diverse range of plant hosts. Even among host generalists, *S. sclerotiorum* is unusually versatile, with a host range that may exceed that of any known fungal phytopathogen^15^. Despite the economic constraints to crop production caused by *S. sclerotiorum* and other broad host-range necrotrophs as crop pathogens, the infection strategies of these species are far from fully elucidated^16^. In particular, their ability to infect a diverse range of plant species is an intriguing area of research^17^. Do these pathogens utilise the same infection strategy on different host species, or are they able to adapt more specifically to their environments? In the latter case, what mechanisms facilitate this flexibility? Though a considerable body of research has examined the determinants of host specificity in biotrophs and specialist necrotrophs^18,19^, these biological questions have not been thoroughly addressed in a broad host range pathogen such as *S. sclerotiorum*. In the case of host generalists, the opposite perspective of “host generality” and its mechanisms may be equally intriguing.

The limited available evidence suggests that the infection strategy of broad host-range necrotrophs may involve a combination of broad-spectrum and host-specific facets. The proteinaceous effector SsSSVP1, which is produced by *S. sclerotiorum*, is a notable example of a broad-spectrum pathogenicity factor^20^. Infection assays demonstrate that mutant SsSSVP1-silenced *S. sclerotiorum* strains are significantly less virulent than the control in both *B. napus* and *Arabidopsis thaliana*, indicating the effector has a significant inter-specific role in the infection process^20^. The metabolite oxalic acid also appears to be employed by *S. sclerotiorum* as a broad-spectrum pathogenicity factor. Infection assays of OA-deficient *S. sclerotiorum* strains on a range of hosts including *G. max*, *P. vulgaris*, *Solanum lycospersicum, B. napus, Helianthus annuus*, and *A. thaliana* resulted in substantially reduced virulence, demonstrating that OA plays an important role in the infection strategy of *S. sclerotiorum* across a wide range of host species^21^. These broad-spectrum pathogenicity factors may contribute to the ability of *S. sclerotiorum* to infect a wide range of plant hosts.

By comparison with broad-spectrum pathogenicity determinants, host-specific infection mechanisms are relatively poorly understood in host-generalist necrotrophs. Gene expression studies suggest that certain pathogenicity factors may be targeted to specific hosts through differential gene expression, though the interactions of these differentially expressed genes have not been characterised. Blanco-Ulate *et al*.^22^ examined differential expression of CAZyme-associated genes in *B. cinerea* on a selection of plant host tissues consisting of lettuce leaves, tomato fruit and grape berries. Pectin-modifying enzymes were more highly expressed on pectin-rich tomato fruit and grape berries than on lettuce leaves, which contain lower levels of pectin^22^. This was thought to suggest a role for differential expression of CAZyme-associated genes in the adaptation of *B. cinerea* to different hosts, possibly contributing to its broad host range.

Host-specific differential gene expression has also been observed in *S. sclerotiorum*. Guyon *et al*.^23^ demonstrated distinct differences in the expression patterns of a selection of 16 effector candidates between the hosts *S. lycospersicum, N. benthamiana* and resistant and susceptible accessions of *A. thaliana*. More broadly, variations in gene expression over time have also been examined in *S. sclerotiorum* using RNA-seq^13^. However, host-specific differential expression across the entire *S. sclerotiorum* genome has not yet been characterised.

This study describes an RNA-sequencing analysis of host-specific differential gene expression in *S. sclerotiorum* during the infection of *L. angustifolius* and *B. napus*, which are the most widely grown broadleaf crops in the West Australian wheatbelt^24^. The primary aim of this study was to identify genes expressed in a host-dependent fashion by *S. sclerotiorum* across the entire genome, in contrast with earlier studies that focussed on specific groups of putative pathogenicity factors^23^. It was hypothesised that host-specific differential gene expression in *S. sclerotiorum* would be observed primarily among putative pathogenicity-related genes, and particularly CAZymes. However, these genes were found to make up only a small portion of all host-specific differentially expressed genes. The diverse set of genes differentially expressed by *S. sclerotiorum in planta* suggest a possible role of differential expression as a means of adaptation to plant defence responses.

## Results

### Adapter trimming and BBSplit read assignment

**Figure 1:**
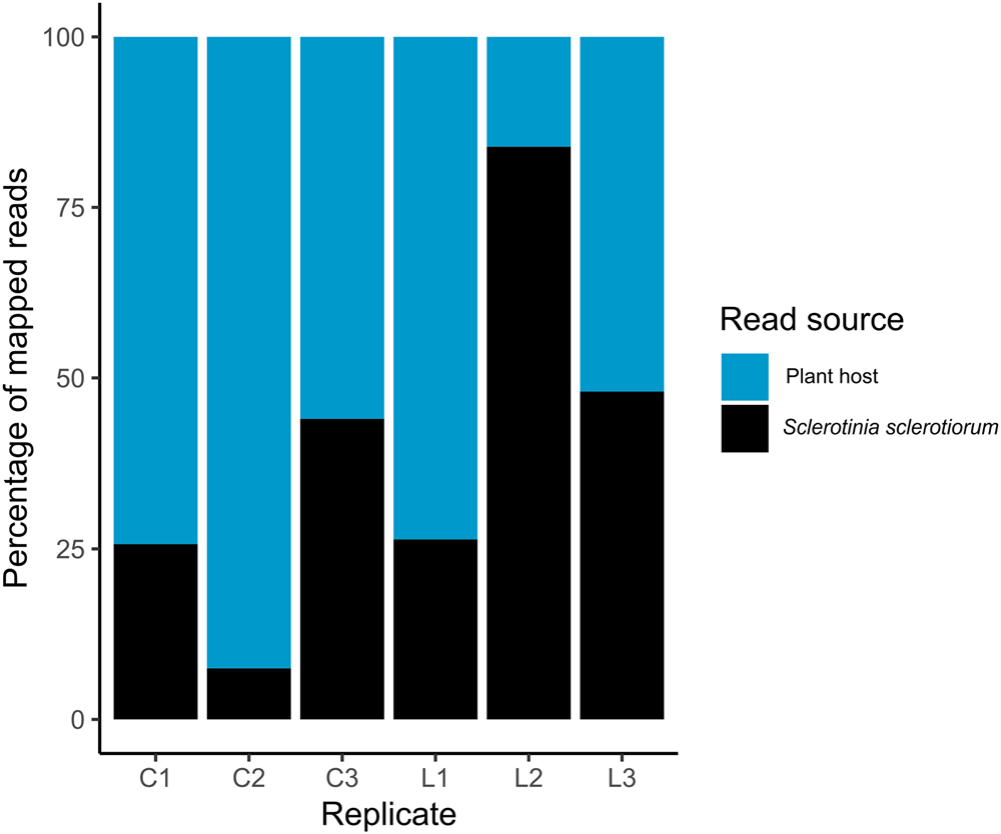
Proportions of reads mapping to *Sclerotinia sclerotiorum* and the plant hosts. Replicates C1–3 correspond to *B. napus*, while replicates L1–3 correspond to *L. angustifolius*. The y axis shows the percentage of mapped reads and bars are separated into blue (mapping to the plant host genome) and black (mapping to the *S. sclerotiorum* genome).

RNA sequencing was used to compare the gene expression of *S. sclerotiorum* during infection of the host species *L. angustifolius* and *B. napus*, along with an *in vitro* control treatment. RNA was extracted from infected plant samples at 3 days post-inoculation (dpi) and sequenced with the Illumina HiSeq platform. The Trimmomatic package was used to remove adapters resulting from the sequencing platform, along with low-quality reads^25^. Trimmomatic read retention appeared to be generally higher in the *in vitro* treatments than in the *in planta* treatments, with respective averages of 95.0% and 92.4% (Table 1). BBSplit was used on the resulting trimmed reads to assign reads to either *S. sclerotiorum* or the respective plant host *(B. napus* or *L. angustifolius*). The proportion of reads mapped to *S. sclerotiorum* varied considerably between the three treatments, from approximately 7.5% (in *B. napus*) to 83.9% (in *L. angustifolius*)(Fig. 1). The mean proportion of reads assigned to the *S. sclerotiorum* genome across all libraries was 39.3%.

**Table 1:** Quality control and assignment rates from Trimmomatic and BBSplit.

Treatment	Replicate	Raw input read pairs	Reads retained by Trimmomatic (%)	*In planta* reads assigned to *S. scler*. by BBSplit (%)	Final read pairs
*In vitro*	1	51,429,980	94.9	NA	47,609,295
*In vitro*	2	43,993,597	94.9	NA	40,898,891
*In vitro*	3	42,595,015	95.2	NA	39,702,901
*B. napus*	1	42,067,707	91	25.7	9,595,914
*B. napus*	2	39,650,442	90.7	7.5	2,630,108
*B. napus*	3	45,312,584	92.9	44	18,079,428
*L. angustifolius*	1	39,982,586	92.7	26.4	9,487,026
*L. angustifolius*	2	40,510,004	92.4	83.9	30,699,672
*L. angustifolius*	3	47,604,827	93.9	48	20,783,466

The fifth column refers to the number of *in planta S. sclerotiorum* reads as a proportion of all *in planta* reads assigned to the host or pathogen by BBsplit.

### Data inspection, quality control and sample clustering analysis

The Limma package in R was used to test differential gene expression across the entire *S. sclerotiorum* genome, making all three possible comparisons between treatments^26,27^. As expected, *S. sclerotiorum-*derived reads were detected in all treatments. Between the three treatments, a total of 10,864 predicted protein-encoding genes were detected at least once, representing approximately 97.6% of the protein-encoding genome. Average gene coverage was greatest in the *in vitro* treatment at approximately 96.9% and slightly lower in the *in planta* treatments at 92.1% in *B. napus* and 94.9% in *L. angustifolius*.

**Figure 2:**
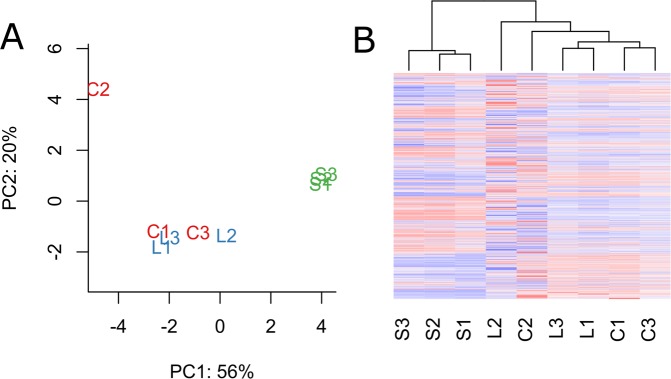
Scatter plot and heatmap based on principal component analysis conducted in Limma based on gene expression levels. The labels ‘C1’, ‘C2’ and ‘C3’ correspond to the three *Brassica napus* infected replicates, the labels ‘L1’, ‘L2’ and ‘L3’ correspond to the three *Lupinus angustifolius* infected replicates, and the labels ‘S1’, ‘S2’ and ‘S3’ correspond to the three *Sclerotinia sclerotiorum in vitro* replicates. (**A**) Text is also coloured in red for *B. napus*, blue for *L. angustifolius* and green for *in vitro* samples. The x-axis is principal component 1 and the y-axis is principal component 2. (**B**) Columns labelled “S” are *in vitro* replicates, “L” are *L. angustifoliu*s and “C” are *B. napus*. Red regions indicate high gene expression levels, which blue regions indicate low gene expression levels. Replicates are clustered according to the hierarchy at the top of the figure.

Principal component analysis plots and heatmaps produced in Limma demonstrated a consistent grouping of the *in vitro* samples (Fig. 2). However, the *L. angustifolius* and *B. napus* samples did not form distinct groups. The Limma PCA plot indicated that one of the *B. napus* replicates was distinct from the others based on its gene expression profile (Fig. 2A). Subsequently, a heatmap produced in Limma indicated that there were in fact two outlying replicates, with one from each *in planta* treatment (Fig. 2B) and statistical adjustments were made for the outliers as described in the materials and methods. In both cases, the *in vitro* samples were relatively consistently clustered.

### Differential expression analysis reveals a set of *Sclerotinia sclerotiorum* genes highly up regulated on two different host species

**Figure 3:**
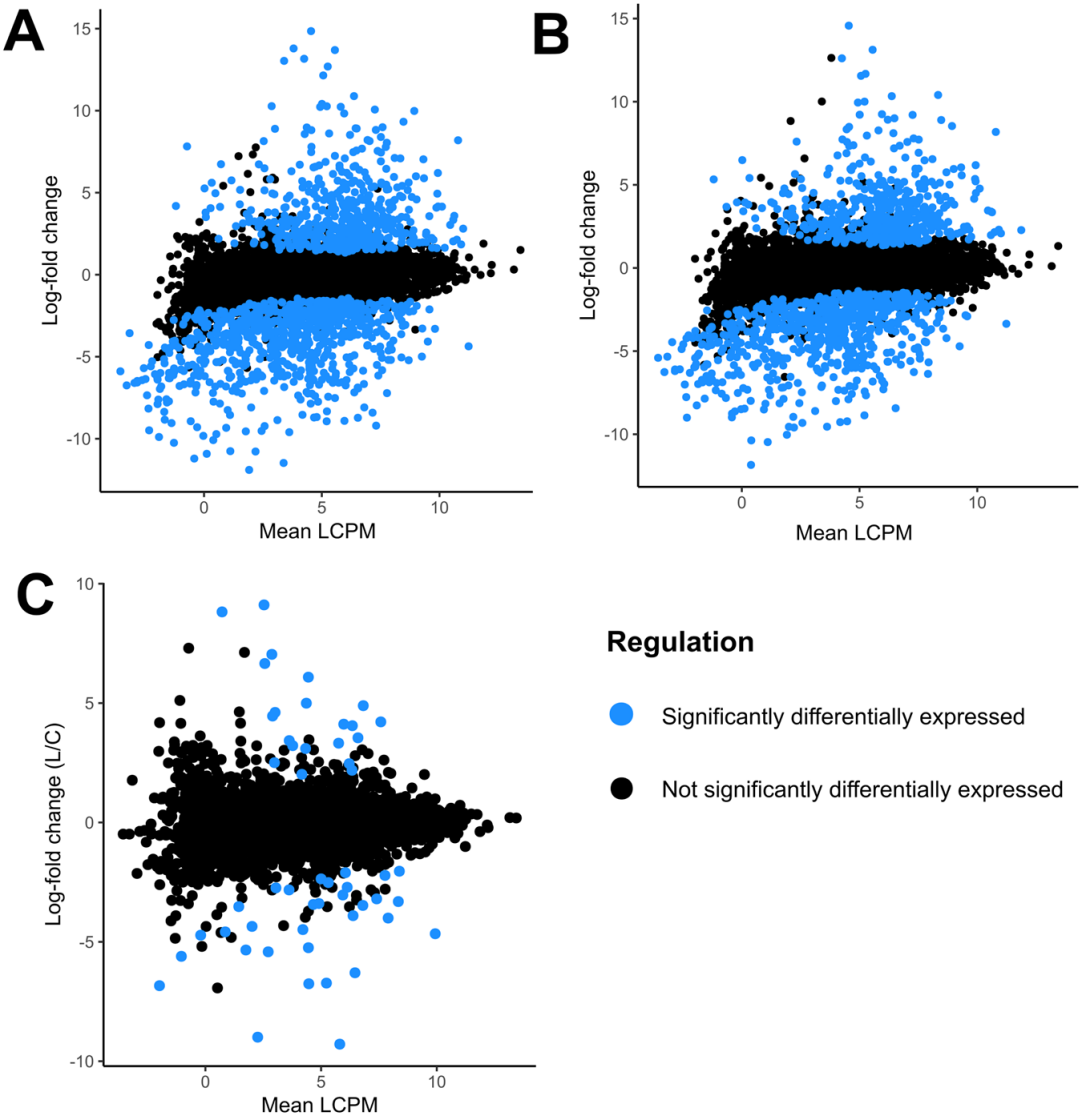
Scatter plots showing log-fold changes in *S. sclerotiorum* gene expression against mean log counts-per-million values for all genes. The x-axis shows the mean log CPM of the respective treatments, while the y-axis show the log fold change between the two treatments. Log-fold changes are *in planta* (*L. angustifolius*) relative to *in vitro* (**A**), *in planta* (*B. napus*) relative to *in vitro* (**B**), *in planta* (*L. angustifolius*) relative to *in planta* (*B. napus*) (**C**).

We hypothesised that the majority of *in planta-*upregulated genes would be induced irrespective of host. To test this hypothesis, we compared the sets of genes upregulated by *S. sclerotiorum* in *B. napus* and *L. angustifolius* relative to the *in vitro* treatment. We did not count genes with less than 1 read per million (RPM) in all three replicates and considered genes differentially expressed if they had a log_2_(fold change) of more than 2 and an adjusted p value of <0.05. In total, 628 genes were significantly upregulated in at least one of the *in planta* treatments relative to *in vitro* (Fig. 3A,B, Supplementary Table 1). The majority of these genes (64%) were upregulated in both *in planta* treatments, supporting our hypothesis. In both *in planta* treatments, the upregulated gene with the largest log-fold change relative to *in vitro* was sscle_15g106470 (14.6 in *B. napus* (p_adj_ = 0.004) and 14.8 in *L. angustifolius* (p_adj_ = 0.003)), which encodes a thioesterase (Table 2). In terms of absolute expression levels, the most highly expressed upregulated gene was sscle_16g108170 in both species, with log-counts-per-million (LCPM) values of 13.7 in *B. napus* (p_adj_ < 0.001) and 13.4 in *L. angustifolius* (p_adj_ < 0.001). This gene encodes for a predicted glycoside hydrolase.

**Table 2:** The top 10 most highly upregulated genes in *B. napus* and *L. angustifolius*, ranked from most highly upregulated to least.

Rank	B. napus					L. angustifolius				
	Gene ID	Pfam domain	LFC	P_adj_	LCPM	Gene ID	Pfam domain	LFC	P_adj_	LCPM
1	sscle_15g106470	Thioesterase domain	14.6	0.004	9.3	sscle_15g106470	Thioesterase domain	14.8	0.003	9.5
2	sscle_05g042570	NmrA-like family	13.1	0.001	10.2	sscle_11g085620	Transferase family	13.8	0.03	8.6
3	sscle_11g085620	Transferase family	12.6	0.05	8.8	sscle_05g042570	NmrA-like family	13.7	0.001	10.2
4	sscle_15g106460	Transferase family	12.6	0.003	8.7	sscle_15g106460	Transferase family	13.2	0.002	8.7
5	sscle_11g085630	Cytochrome P450	11.7	0.004	9.8	sscle_07g060710	FAD binding domain	13	0.023	5.7
6	sscle_15g106450		11.6	0.002	9	sscle_11g085630	Cytochrome P450	12.7	0.002	9.6
7	sscle_08g067130	Cytochrome P450	10.4	6.30E-07	9.4	sscle_15g106450		12.1	0.001	9.2
8	sscle_15g106500		10.3	1.30E-04	10.1	sscle_15g106500		10.9	8.00E-04	10.1
9	sscle_07g060710	FAD binding domain	10	0.087	8.6	sscle_08g068200	Chitin recognition protein	10.4	0.001	8.4
10	sscle_15g106490	FAD binding domain	10	0.001	8.6	sscle_02g018820	Glycosyl hydrolases family 28	10.3	0.002	7.1

“LFC” is log-fold change (base 2), “LCPM” is log-counts-per-million *in planta* (base 2).

Necrotrophic phytopathogens may utilise a combination of secreted effectors, secondary metabolites and CAZymes to facilitate infection^3,20,28,29^. Therefore, we hypothesised that genes within these functional categories would be over-represented among genes upregulated *in planta* relative to *in vitro*. To test this hypothesis, a Chi-square test of independence (α = 0.05) was conducted to assess the association between upregulation and gene function, for the categories of CAZyme-associated genes, secondary metabolite-associated genes and putative effector-encoding genes. Using DBCan2, we identified 173 CAZymes in the *S. sclerotiorum* genome. Using AntiSMASH, we identified 87 secondary metabolite biosynthesis clusters containing 1,630 genes. To identify effector genes, we used the 70 effector predictions from Derbyshire *et al*. (2017).

Gene function and upregulation were found to be associated (X^2^ = 311.99, df = 3, p < 0.001). All three mentioned gene categories were upregulated more frequently than expected by chance alone. Notably, 98 CAZyme-encoding genes and 20 secondary-metabolite associated genes were upregulated, which is considerably greater than the expected frequencies of approximately 27.7 and 9.5 respectively.

### Expression analysis shows that *Sclerotinia sclerotiorum* differentially regulates a subset of 53 genes on two different host species

Expression analysis conducted in Limma showed that *S. sclerotiorum* differentially regulated a subset of 53 genes between the two host species. These included 21 genes upregulated in *L. angustifolius* relative to *B. napus*, with the remaining 32 downregulated (up-regulated on *B. napus* relative to *L. angustifolius*) (Fig. 3C). Again, we did not count genes with less than 1 read per million (RPM) in all three replicates and considered genes differentially expressed if they had a log_2_(fold change) of more than 2 and an adjusted p value of <0.05.

Four differentially expressed genes had log-fold changes of greater than 8, namely sscle_02g022130 (LFC = 9.3, p_adj_ < 0.001) and sscle_04g037240 (LFC = 9.0, p_adj_ = 0.002), which were up-regulated on *B. napus*, and sscle_10g076570 (LFC = 9.1, p_adj_ = 0.004) and sscle_15g106670 (LFC = 8.8, p_adj_ = 0.004), which were up-regulated on *L. angustifolius* (Table 3). The differentially expressed genes with the greatest absolute expression *in planta* were sscle_10g079920 (LFC = 4.7 p_adj_ < 0.001) and sscle_08g067130 (LFC = 3.3, p_adj_ = 0.002), with LCPM values of approximately 13.7 and 13.0 respectively in *B. napus* (Table 3, Fig. 3C); these genes were both up-regulated on *B. napus* relative to *L. angustifolius*. The gene sscle_10g079920 encodes a carbon-nitrogen hydrolase Pfam domain, while sscle_08g067130 encodes a cytochrome P450 domain.

**Table 3:** All genes significantly differentially expressed by *S. sclerotiorum* between the *in planta* treatments.

Gene ID	Pfam domain	Upreg. in:	Abs. LFC	*B. napus* CPM	*L. ang*. CPM	P_adj_
sscle_05g040340	Metallo-beta-lactamase	C	6.8	670.1	4.6	5.10E-06
sscle_05g047240	Serine hydrolase FSH	C	3.5	63.5	9.2	5.00E-02
sscle_02g012440	Major facilitator superfamily	C	3.9	75.6	8.3	4.40E-03
sscle_05g040320	Major facilitator superfamily	C	5.3	33.5	0.7	6.50E-04
sscle_04g037240	Alcohol dehydrogenase, N-terminal	C	9	159.7	0.2	1.80E-03
sscle_08g062510	Methyltransferase type 11	C	4	1701.5	90.6	2.70E-04
sscle_01g006290	Cytochrome P450	C	4.1	58.1	628.4	2.50E-04
sscle_03g026490	NAD-dependent epimerase/dehydratase, N-terminal domain	C	4.4	15.9	0.7	3.60E-03
sscle_06g048630	V-ATPase proteolipid subunit C-like domain	L	2.5	14.1	47.4	2.80E-02
sscle_07g059310	DSBA-like thioredoxin domain	L	2.5	105.5	20.1	2.60E-02
sscle_09g073010	TPMT family	C	6.3	1844.3	24.3	2.70E-05
sscle_02g021040	FAD linked oxidase, N-terminal	C	2.2	208.8	612.9	3.80E-02
sscle_05g047210	Major facilitator superfamily	C	3.2	169.2	23.5	3.80E-03
sscle_10g079920	Carbon-nitrogen hydrolase	L	4.7	12795.5	481.1	1.00E-04
sscle_10g079370	Glycoside hydrolase family 3 C-terminal domain	L	3.4	6.3	43.1	5.30E-03
sscle_04g036270	Acetoacetate decarboxylase	L	3.5	106	709.1	1.50E-03
sscle_02g018820	Glycoside hydrolase, family 28	C	7	11.2	148.3	2.90E-03
sscle_03g022380	NADH:flavin oxidoreductase/NADH oxidase, N-terminal	C	2.7	363.5	43.2	8.20E-03
sscle_03g026590	Glycoside hydrolase family 12	L	4.6	23.1	95.3	2.30E-02
sscle_05g046770	GNAT domain	L	4.5	166.6	6.1	7.60E-04
sscle_16g108230	Carbon-nitrogen hydrolase	C	6.7	841.3	7.3	2.00E-06
sscle_05g040330	Zn(2)-C6 fungal-type DNA-binding domain	L	5.4	62.8	1.3	1.40E-04
sscle_07g058260	Short-chain dehydrogenase/reductase SDR	L	3.5	7.5	0.8	3.20E-02
sscle_08g067140	Tannase/feruloyl esterase	L	2	25.2	101.5	3.60E-02
sscle_10g076570	Tannase/feruloyl esterase	C	9.1	0.7	207.5	3.80E-03
sscle_10g074860	FAD-binding domain	C	2.7	39.5	7.4	3.80E-02
sscle_05g046750	Hypoxia induced protein, domain	L	2.2	882.8	172.1	2.50E-03
sscle_01g005000	Glutathione S-transferase, N-terminal	L	5.2	511.1	11.8	1.60E-05
sscle_04g033880	Cytochrome P450	C	4.2	283	3196	2.50E-03
sscle_12g089000	Alpha/beta hydrolase fold-1	L	5	32.7	328.1	3.60E-03
sscle_08g067130	Cytochrome P450	L	3.3	8287.7	636.4	2.20E-03
sscle_15g105450	Pirin, N-terminal domain	C	3.4	134.4	18.3	3.50E-02
sscle_15g106860	Isochorismatase-like	L	4.6	8.4	0.6	2.60E-02
sscle_03g023780	Carboxylesterase, type B	C	4.1	107.2	559	4.40E-03
sscle_07g058990	Glycoside hydrolase family 16	C	5.6	0.2	4.00E-02	3.40E-02
sscle_02g022130	DJ-1/PfpI	L	9.3	1433.9	3.3	1.30E-04
sscle_05g047220	Phosphatidylethanolamine-binding protein PEBP	C	2	245.9	78.5	3.50E-02
sscle_13g092350		L	3	186	23	7.90E-06
sscle_07g059700		C	6.1	5.9	193.7	2.60E-04
sscle_14g098740		C	4.9	84.8	1184.5	1.80E-03
sscle_03g024860		C	4.5	3.3	51.8	1.80E-03
sscle_04g033890		L	3.1	13.5	77.5	2.40E-03
sscle_03g030870		C	6.7	0.2	88.6	2.50E-03
sscle_10g079930		C	2.8	60.3	8.3	3.60E-03
sscle_15g106670		C	8.8	0	55.3	3.60E-03
sscle_13g096350		C	2.5	6.9	41.9	4.10E-03
sscle_08g064180		C	2.4	237.9	52.6	7.10E-03
sscle_05g045300		C	3.3	63	362.4	8.60E-03
sscle_05g047230		L	2.1	54.7	12.5	1.70E-02
sscle_05g042600		L	3.4	99.8	13.7	2.60E-02
sscle_07g061670		L	4.7	4.4	0.3	2.80E-02
sscle_02g022120		C	6.8	0.3	5.40E-03	3.50E-02
sscle_02g013360		C	3.2	6.8	48.9	3.80E-02

“Abs. LFC” refers to the magnitude of log-fold change in gene expression between the *in planta* treatments (base 2), “LCPM” refers to the gene expression level in log-counts-per-million (base 2). “L” refers to the *L. angustifolius* treatment, “C” refers to the *B. napus* treatment.

**Figure 4:**
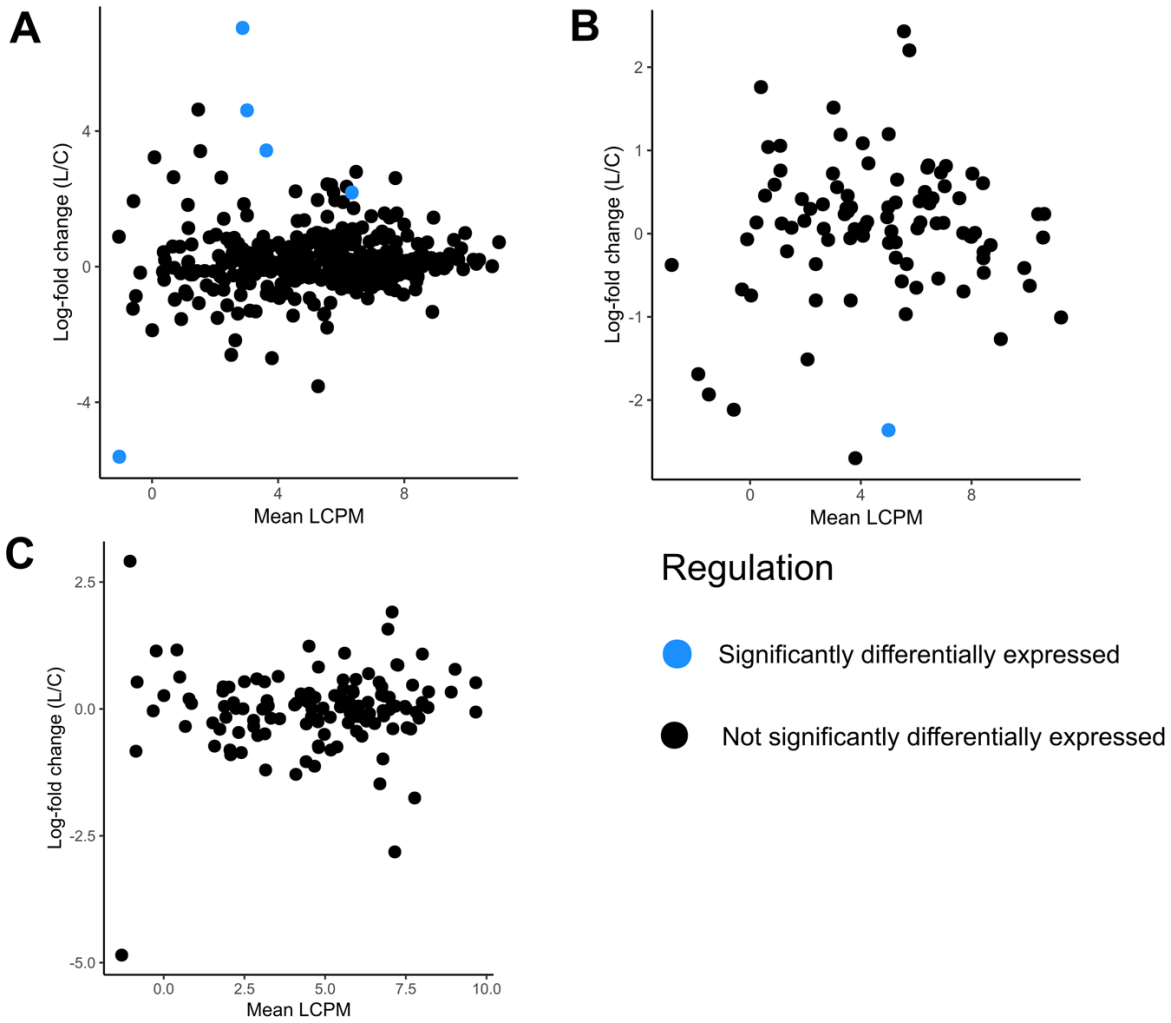
Scatter plots showing log-fold changes in *S. sclerotiorum* gene expression against mean log counts-per-million values between the two *in planta* treatments. The x-axis shows the mean log CPM of the respective treatments, while the y-axis shows the log fold change of gene expression in *L. angustifolius* relative to *B. napus*. The gene subsets are predicted secreted CAZymes (**A**), putative effectors (**B**), and putative secondary metabolites (**C**). No secondary metabolites were significantly differentially expressed.

The *in planta* differentially expressed genes included secreted CAZymes and a solitary putative effector (Fig. 4). The five differentially expressed CAZymes included three glycoside hydrolases, a pectin lyase fold and a vanillyl-alcohol oxidase (Fig. 4A). All but one of these CAZymes were upregulated in *L. angustifolius* relative to *B. napus* (Fig. 4B). The differentially expressed putative effector had no associated GO terms. None of the putative secondary metabolite genes were differentially expressed on the two different hosts (Fig. 4C).

### Quantitative polymerase chain reaction supports the hypothesis differential expression of a subset of genes between host environments

To further test the hypothesis that some genes are specifically up-regulated in *S. sclerotiorum* in response to host environment, we performed quantitative PCR (qPCR). The 15 genes we subjected to qPCR are listed in Supplementary Table 2, along with their expression profiles in the RNA sequencing analysis. These genes included nine that, according to the RNA sequencing analysis, were significantly up-regulated in *B. napus* relative to *L. angustifolius* and six vice versa, and two that were up-regulated in both *in planta* samples relative to *in vitro*.

**Figure 5:**
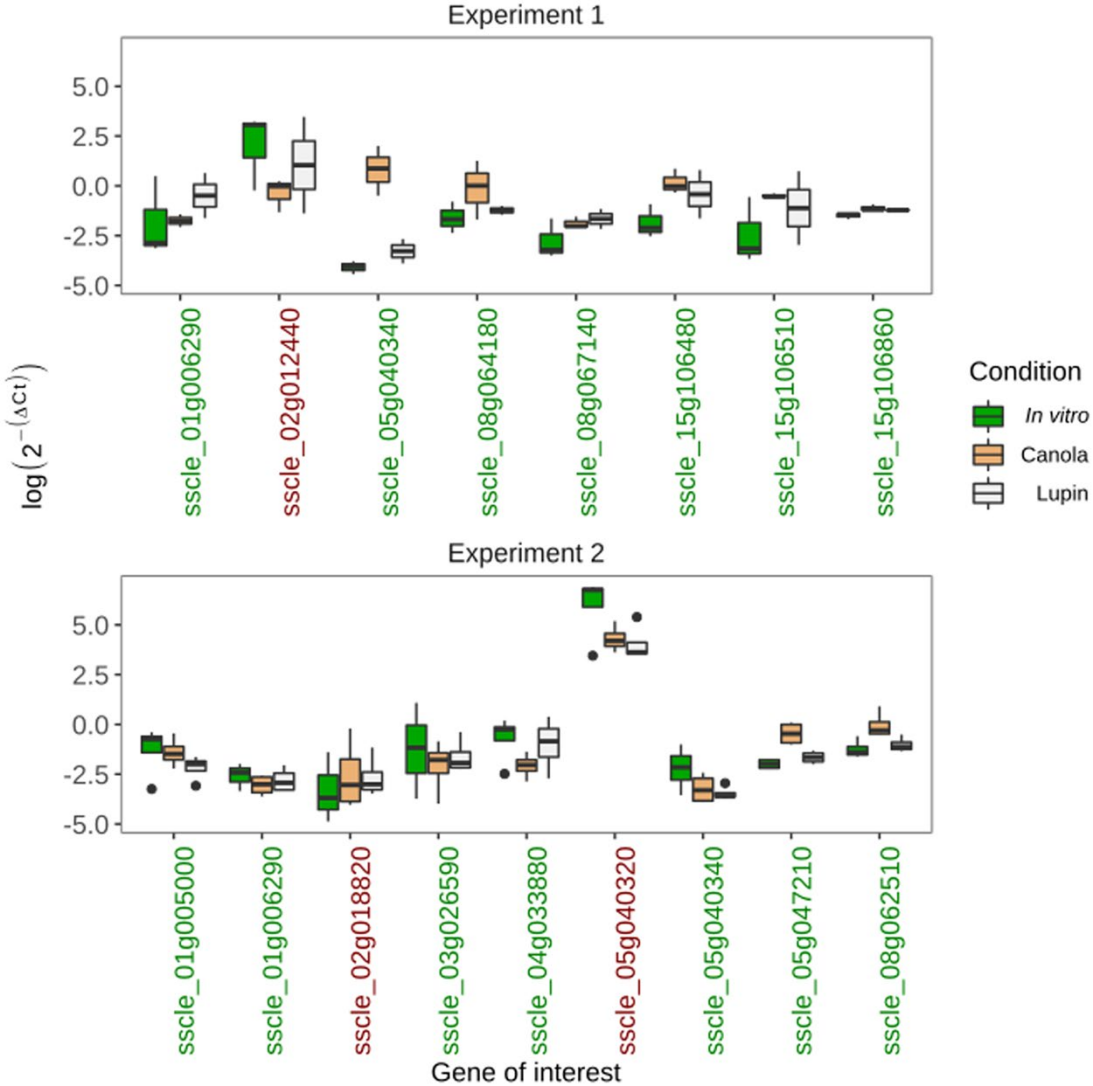
Quantitative PCR analysis of selected genes. The results of two experiments are presented. Experiment 1 used the same methodology as the RNA sequencing analysis to generate samples. Experiment 2 used the methodology of Seifbarghi *et al*. (2017)^13^. The y axis shows the inverse ∆Ct value relative to the housekeeping gene *Sclerotinia sclerotiorum β-tubulin*. The x axis shows the names of genes included in the analysis. The gene names coloured in green correspond with the RNA sequencing analysis, whereas those in red do not. The thick horizontal black lines represent median values. The boxes and whiskers represent interquartile range and the black points represent outliers. For most of the genes, we were concerned with differential expression between *Lupinus angustifolius* and *Brassica napus*, as this was the primary hypothesis we were testing. We also selected the two genes sscle_15g106480 and sscle_15g106510 as they were significantly up-regulated on both *B. napus* and *L. angustifolius* relative to *in vitro*.

Our first experiment (experiment 1) was a set of duplicate (*L. angustifolius*) and triplicate (*B. napus* and *in vitro*) samples independent from the RNA sequencing samples but generated in the same fashion. Out of the eight genes analysed in this experiment, seven showed expression changes in the same direction as they did in the RNA sequencing analysis (Fig. 5). The two genes sscle_05g040340 and sscle_08g06410 in particular showed convincing evidence of up-regulation on *B. napus* relative to *L. angustifolius* as they both exhibited non-overlapping standard errors between the two samples. According to the RNA sequencing data, the genes sscle_15g106480 and sscle_15g106510 were up-regulated in both *L. angustifolius* and *B. napus* relative to *in vitro*. Our qPCR assay agreed with this, however, the standard error between the two replicates was high for the *L. angustifolius* samples and it overlapped with that of the *in vitro* samples. The gene sscle_02g012440 did not exhibit the same expression profile as was observed in the RNA sequencing data. In the RNA sequencing analysis, this gene was up-regulated in *B. napus* relative to *L. angustifolius*. However, in our qPCR analysis, we observed the opposite.

To formally test the hypothesis of differential expression between conditions, we performed Welch’s t-test for *B. napus* and *L. angustifolius* comparisons, and analysis of variance when considering all three samples, *B. napus*, *L. angustifolius* and *in vitro*. For a single gene, sscle_05g040340, we could confidently accept the alternate hypothesis of differential expression between two samples based on qPCR data (*B. napus*: mean 2^−∆Ct^ = 3.46, standard deviation +/−3.51; *L. angustifolius*: mean 2^−∆Ct^ = 0.045, standard deviation +/−0.035. Welch’s t = 4.3, df = 2.89, P = 0.025). We infer that the general agreement between qPCR and RNA sequencing data suggests that the RNA sequencing results would likely be similar across further replicated data sets. However, we can only confidently state that a single candidate gene has strong evidence for up-regulation on *B. napus* relative to *L. angustifolius*.

In our second experiment (experiment 2), we infected detached leaves using mycelial matts. This experiment was replicated four times per condition. We tested nine genes in this experiment, two of which were also included in experiment 1 (the genes sscle_01g006290 and sscle_05g040340). Overall, seven out of nine genes exhibited changes in expression between *B. napus* and *L. angustifolius* in the same direction as observed in the RNA sequencing data; these included the two genes shared between experiment 1 and experiment 2. However, in contrast to experiment 1, in experiment 2, these two genes exhibited overlapping standard errors of their mean 2^−∆Ct^ values. Four out of the seven genes had non-overlapping standard errors, suggesting strong differential expression. These included sscle_08g062510, sscle_05g047210 and sscle_01g005000, which were up-regulated on *B. napus* and sscle_04g033880, which was up-regulated on *L. angustifolius*. These all agreed with the direction of change in expression in the RNA sequencing analysis. The two genes that disagreed with the RNA sequencing data were sscle_02g018820 and sscle_05g040320. The former was expressed to a greater level on *B. napus* in the qPCR data and *L. angustifolius* in the RNA sequencing data. The latter showed only a negligible increase in expression from *L. angustifolius* to *B. napus*, whereas in the RNA sequencing analysis this difference was substantial.

To formally test the hypothesis of differential expression between conditions in experiment 2, we performed Welch’s t-tests. For two genes, sscle_08g062510 and sscle_05g047210, we could relatively confidently accept the alternate hypothesis of differential expression between the two samples based on qPCR data (sscle_08g062510: *B. napus*: mean 2^−∆Ct^ = 1.15, standard deviation = 0.9; *L. angustifolius*: mean 2^−∆Ct^ = 0.38, standard deviation = 0.15. Welch’s t = 2.59, df = 4.71, P = 0.052. sscle_05g047210: *B. napus*: mean 2^−∆Ct^ = 0.7, standard deviation = 0.3; *L. angustifolius*: mean 2^−∆Ct^ = 0.19; standard deviation = 0.06. Welch’s t = 3.71, df = 4.66, P = 0.016). We infer that the general agreement between qPCR and RNA sequencing data suggests that the RNA sequencing results would likely be similar across further replicated data sets. However, we can only confidently state that two candidate genes have strong evidence for up-regulation on *B. napus* relative to *L. angustifolius*.

## Discussion

Necrotrophic phytopathogens such as *S. sclerotiorum* are known to utilise a variety of pathogenicity factors to facilitate infection, including CAZymes, proteinaceous effectors and secondary metabolites^7,20,30^. Existing evidence on pathogenicity factors in *S. sclerotiorum* consists of a combination of qPCR and RNA-seq based expression studies, knockout assays and biochemical studies focussing on select genes^13,20,23^. These studies include investigations of the broad-spectrum effector SsSSVP1, the metabolite oxalic acid, and a group of 16 putative effectors^20,21,23^. To our knowledge, this study is the first to utilise RNA sequencing to investigate host-specific gene expression across the entire *S. sclerotiorum* transcriptome, including a wide range of predicted pathogenicity factors. Many of the upregulated genes do not appear to have been examined by previous host-specific expression studies, with the exception of a selection of putative effectors^23^.

According to the results of a Chi-square test, secreted CAZymes, secondary metabolites and putative effectors were collectively upregulated *in planta* relative to *in vitro* (X^2^ = 311.99, df = 3, p < 0.001). These results suggest that these groups of genes are indeed involved in the infection strategy of *S. sclerotiorum*. Secondary metabolites, effectors and CAZymes have previously been described in more than one necrotrophic species. Notable examples include the secondary metabolite botrydial in *B. cinerea*^7^, the effector SsSSVP1 in *S. sclerotiorum*^20^ and a variety of predicted CAZymes including cellulases, pectinases and xylanases in *S. sclerotiorum*^31^. Consequently, the upregulation of these gene categories by *S. sclerotiorum* is consistent with earlier work implicating them in the infection process.

Among the main groups of pathogenicity factors, CAZymes formed the largest group of upregulated genes. A total of 98 secreted CAZymes were upregulated by *S. sclerotiorum in planta* relative to *in vitro* in at least one species. A variety of CAZymes have been predicted in the *S. sclerotiorum* genome, which include pectinases, glucanases and cellulases^4^. Many of these genes are thought to encode plant cell wall-degrading enzymes (PWDEs), which are involved in degrading the diverse structural molecules of plant cell walls such as cellulose, hemicellulose and pectin^4^. The upregulation of secreted CAZymes *in planta* is therefore entirely consistent with their theoretical role in the necrotrophic infection strategy.

Putative secondary metabolites were also upregulated by *S. sclerotiorum in planta* relative to the *in vitro* treatment. Though some secondary metabolites are thought to act as secreted toxins in other fungal necrotrophs^32–35^, they appear to be poorly understood in *S. sclerotiorum*. By comparison, the secondary metabolites botrydial and botcinic acid are known to be important virulence factors in *B. cinerea*^7^. A variety of polyketide synthases (PKS) and non-ribosomal peptide synthases (NRPS) are encoded within the *S. sclerotiorum* genome, suggesting that the fungus may produce polyketides and non-ribosomal peptides as secondary metabolites^13^. These compounds are known to be among the phytotoxic secondary metabolites secreted by the closely related *B. cinerea*^28^.

Two PKS-encoding genes, namely sscle_15g106480 and sscle_15g106510, were upregulated *in planta* in this study in at least one plant treatment. The orthologues of these genes in *B. cinerea* are involved in the production of botcinic acid, which is an important pathogenicity factor in the species^7^. Limited evidence is available regarding the production of botcinic acid by *S. sclerotiorum*, and the compound has not been characterised in this pathogen^7^. Seifbarghi *et al*.^13^ found that only sscle_15g106480 was upregulated by *S. sclerotiorum* during infection of *B. napus*, and suggested that the pathogen is unlikely to produce botcinic acid because both sscle_15g106480 and sscle_15g106510 are required for synthesis^7^. In this study, both genes were upregulated in *L. angustifolius*, which is consistent with the synthesis of botcinic acid or a related compound (LFC = 3.8, p_adj_ < 0.001, LFC = 9.2, p_adj_ < 0.001 respectively). However, in *B. napus* these genes were significantly downregulated relative to *in vitro*, which suggests that the role of botcinic acid may be host specific (LFC = −3.3, p_adj_ < 0.001, LFC = −5.8, p_adj_ < 0.001 respectively). Perhaps the downregulation of these genes in *B. napus* suggests that the plant defence responses have been largely overcome, negating the need to secrete botcinic acid. However, it is difficult to draw any convincing conclusions given the advanced stage of sample infection and the absence of evidence on the production of botcinic acid by *S. sclerotiorum*^7^.

An alternative explanation for the discrepancies between Seifbarghi *et al*.’s and our observations is that we took samples at different time points from plants inoculated with different methods. In Seifbarghi *et al*., a matt of mycelium was used to infect detached *B. napus* leaves and in our study stems were infected with agar plugs. Our infection samples were taken at 3 DPI as opposed to the 6 point time course taken up to a maximum of 2 DPI in Seifbarghi. Furthermore, Seifbarghi *et al*. focused on the reference strain of *S. sclerotiorum*, 1980, which is genotypically divergent from the Western Australian strain used in our study. Further research is needed to determine differences in gene content and expression between isolates of *S. sclerotiorum* at different time points in different hosts.

The primary aim of this study was not to investigate gene expression *in planta* relative to *in vitro*, but to examine differential gene expression as a response to different plant hosts. In comparison to the 628 genes upregulated *in planta*, only 53 genes were differentially expressed between the two plant hosts (*B. napus* and *L. angustifolius*) (Fig. 3c). The relatively small number of host-specific differentially expressed genes (DEGs) is consistent with the PCA and hierarchical clustering analysis, which consistently identified differences between the *in vitro* and *in planta* treatments but failed to identify distinct *B. napus* and *L. angustifolius* treatments. One explanation for the small number of significantly differentially expressed genes is that the environments presented by the plant hosts to *S. sclerotiorum* are more similar to one another than they are to the *in vitro* control. Alternatively, perhaps the number of differentially expressed genes does not accurately reflect the differences between the plant hosts, and both species present biological cues that prompt upregulation of broad-spectrum virulence factors *in planta*. Some effectors could potentially be upregulated in all plant hosts if they interact with highly conserved plant genes. The *S. sclerotiorum*-derived effector SsSSVP1 is a potential example of this effect as it is thought to act on mitochondrial respiration^20^. Accordingly, this gene was not significantly differentially expressed between the plant hosts in this study.

It is also worth considering the possible influence of the late sampling stage on the results. The infection process of *S. sclerotiorum* is thought to be a two-step process, in which an early biotrophic phase is followed by a later necrotrophic phase^13,14^. *S. sclerotiorum* is thought to secrete oxalic acid and possibly other compounds as “compatibility” factors to subvert host defence responses during the early biotrophic stage of infection^13,31^. Differential expression of genes associated with these compatibility factors has been suggested as a form of host-specific adaptation in *S. sclerotiorum*^13^. Any compatibility factors differentially expressed during the earlier biotrophic stage of infection would not necessarily be represented in this study, as the biotrophic phase of infection is thought to occur 12–24 hours post-inoculation (hpi), which is considerably earlier than the 72 hpi time point used in this study^13^.

Seifbarghi *et al*.^13^ suggested two products in particular as potentially host-specific compatibility factors, neither of which were shown to be differentially expressed in this study. The first of these is secreted integrin-like protein SSITL, which suppresses jasmonic acid/ethylene-mediated resistance responses^13,36^. The associated gene sscle_08g068500 was not significantly differentially expressed by *S. sclerotiorum* between the two plant hosts. The second of these candidate host-specific compatibility factors is the chorismate mutase SScm1^13^. This enzyme has not been characterised, but is thought to be secreted by *S. sclerotiorum* to suppress salicylic acid synthesis, which is a key plant defence response^37^. The gene responsible for SScm1 has not been positively identified, though the gene sscle_16g111080 matches the domain^38^. This gene was not significantly differentially expressed by *S. sclerotiorum* between *L. angustifolius* and *B. napus*, suggesting that it is not involved in the transcriptional adaptation of *S. sclerotiorum* to these host species in the late stages of infection. However, we emphasise here that the late time points of our study preclude a comprehensive overview of gene expression at all stages of infection in both hosts. The discrepancies between the work of Seifbarghi *et al*. and us may be better explained by methodological differences. Additionally, in our experiment, one of the samples generated from infected *B. napus* tissue produced relatively few reads mapping to the *S. sclerotiorum* genome. This low abundance of mapped reads led to significant divergence of the replicate from others in the dataset. This discrepancy could have also affected inference from our differential expression analysis.

The production of salicylic acid may be vulnerable to interference at more than one stage. Earlier research in *Ustilago maydis* suggests that SA production could be reduced by fungal secretions of chorismate mutase, which could potentially be produced by the gene sscle_16g111080^38,39^ (Table 3). Another potential target is isochorismate, which follows chorismate in one of the SA synthesis pathways in plants^40^. The gene sscle_15g106860, which has an isochorismatase-like Pfam domain, was significantly upregulated in *L. angustifolius* relative to *B. napus* (LFC = 4.6, p_adj_ = 0.026). Isochorismatases catalyse the breakdown of the isochorismate into smaller by-products, potentially diverting isochorismate from subsequent stages of the synthesis process. This may suggest that sscle_15g106860 is involved in the disruption of SA synthesis in a host-specific fashion.

A total of five predicted CAZyme-associated genes were differentially expressed by *S. sclerotiorum* between the two host species. Of these five genes, all but one encoded for glycoside hydrolases. Glycoside hydrolases are a highly diverse group of enzymes that catalyse the hydrolysis of glycosidic bonds between carbohydrate subunits, reflecting the structural variety of their substrates^41^. The remaining gene, sscle_02g021040, encoded a predicted vanillyl alcohol oxidase and was upregulated in *L. angustifolius* relative to *B. napus* (LFC = 2.2, p_adj_ = 0.038). It is difficult to speculate on the role of this gene, as the role of vanillyl alcohol oxidases in fungi is generally poorly understood^42^.

The differentially expressed glycoside hydrolases (GHs) may hint at differences between the composition of plant cell walls. The four predicted GHs all belonged to different GH families, without any obvious pattern. The families GH12, GH3, GH28 and GH16 are associated with the degradation of cellulose, beta-glycans, pectins^30^ and hemicellulose respectively^43^ (Table 3). These substrates are all well-known cell wall components across the plant kingdom, which raises the question of why certain CAZymes would be upregulated in specific hosts^44^. One possibility is that these differentially expressed CAZymes act on variants of cell-wall components peculiar to specific plant hosts. The cell-wall components hemicellulose and pectin are known to vary considerably in chemical composition and structure between plant species, which provides some support for this proposition^45^. This diversity is further reflected in the *S. sclerotiorum* genome, which contained some 215 GH-associated genes according to HMMER predictions conducted in DbCan2. However, it is difficult to be specific about the roles of these host-specific CAZyme-associated genes without functional characterisation. The few CAZyme genes characterised by Li *et al*.^6^ do not correspond to the gene models developed by Amselem *et al*.^4^ or Derbyshire^38^, and consequently it is unclear whether these CAZymes correspond to differentially expressed genes in this study. Despite the inability to assign specific roles to CAZyme-associated DEGs, the results of this study indicate a role for gene expression in the adaptation of *S. sclerotiorum* to different host substrates.

Host-specific expression of effector candidate genes was more restricted than may be expected based on previous research. Of the effector candidates predicted by Derbyshire *et al*.^38^ and Amselem *et al*.^4^, only one effector candidate gene, namely sscle_08g064180, was significantly differentially expressed between the two host species. This gene was upregulated in *B. napus* relative to *L angustifolius* (LFC = 2.4, p_adj_ = 0.007)(Table 3). By contrast, a previous quantitative PCR study of a select group of 16 effector candidates in *S. sclerotiorum* produced five separate groups of effector candidate genes, each with varying patterns of expression across time points and species^23^. The differentially expressed putative effector sscle_08g064180 is predicted to have a coiled coil domain, though there appears to be little evidence regarding the role of these domains in effectors.

Though secreted CAZymes, effectors and secondary metabolites play a heavily emphasised role as pathogenicity factors in the necrotrophic infection strategy, they may not tell the full story of host-specific gene expression. Previous research into host-specific gene expression in fungal necrotrophs has focussed on pathogenicity factors such as effector candidates and CAZymes to the exclusion of others^22,23^. As a result, the broader story of host-specific gene expression is poorly understood. In this study, the 53 genes differentially expressed by *S. sclerotiorum* in a host-specific fashion included only six encoding for CAZymes, putative effectors and secondary metabolites, which are often considered important factors in molecular plant-necrotroph interactions^7,20,22^. Investigation of the remaining genes is necessary to provide a broader picture of host-specific *S. sclerotiorum*-host interactions.

A total of 47 host-specific DEGs were not associated with CAZymes, putative effectors or secondary metabolites. What then, is the function of this surprisingly large group of genes? One possibility is that some of the host-specific DEGs are involved in the detoxification of phytotoxic compounds secreted by the plant host. *S. sclerotiorum*, for example, is known to metabolise phytoalexins produced by crucifer hosts during infection, effectively converting them into smaller non-toxic molecules^8^. Similarly, the fungal phytopathogen *Grosmannia clavigera*, which infects pines, is thought to utilise a variety of mechanisms to detoxify host-produced terpenes^9^. These mechanisms may include modification of the terpenes to less toxic molecules and transporter-based efflux^46^. Flexible detoxification mechanisms in *S. sclerotiorum* could contribute to its remarkable adaptability to diverse host species.

Several host-specific DEGs are possible candidates for involvement in the detoxification of plant-produced secondary metabolites. Of particular interest are the three host-specific cytochrome P450-encoding DEGs, sscle_01g006290 (LFC = 4.1, p_adj_ < 0.001), sscle_04g033880 (LFC = 4.2, p_adj_ = 0.002) and sscle_08g067130 (LFC = 3.3, p_adj_ = 0.002)(Table 3). Cytochrome P450s (CYPs) are a broad group of genes that are commonly involved in metabolism pathways, and are particularly well known for the modification and degradation of xenobiotic compounds^47,48^. Examples of CYPs in fungi include the CYP53 family, which are known to be involved in detoxifying isoeugenol, benzoic acid, and other phytotoxic compounds produced by plant hosts in *Cochliobolus lunatus*^49^. The differential expression of CYPs by *S. sclerotiorum* in a host-specific manner, with log-fold changes as great as 4.2, suggests that these genes may be expressed in response to species-specific plant-derived compounds. Given the known role of CYPs in the detoxification of plant secondary metabolites by phytopathogens, it is quite possible that some of the CYP-associated DGEs may be involved in detoxification.

A variety of other host-specific DGEs could be involved in fungal detoxification of plant molecules. The genes sscle_08g067140 (LFC = 2.0, p_adj_ = 0.036) and sscle_10g076570 (LFC = 2.5, p_adj_ = 0.004) appear to encode for tannases, which degrade tannins (Table 3). Plant-produced tannins are known to be toxic to fungal phytopathogens such as *Crinipellis perniciosa* and *Pythium insidiosum*^50,51^. Consequently, it appears likely that the differential expression of tannase-encoding genes indicates detoxification of plant-produced tannins. Interestingly, sscle_08g067140 and sscle_10g076570 were upregulated in different host species (*L. angustifolius* and *B. napus* respectively), suggesting that the plant hosts produce different forms of tannin.

The metallo-beta-lactamase encoding gene sscle_05g040340 may also play a role in the detoxification of plant secondary metabolites by *S. sclerotiorum* (LFC = 6.8, p_adj_ < 0.001)(Table 3). In other species, metallo-beta-lactamases are well-known as metabolic enzymes of xenobiotic compounds. Notably, the New Delhi metallo-beta-lactamase-1 enzyme produced by several human-pathogenic bacteria is known to be involved in the metabolism of antibiotics, resulting in antibiotic resistance^52^. In fungi, these metallo-beta-lactamases are thought to act on a range of lactams not restricted to beta-lactams^10^. Lactones, which are chemically related to lactams, are known to be produced by plants^53^. Several synthetic lactones have been shown to have antifungal effects on fungal pathogens including *B. cinerea, Penicillium citrinum*, and several *Aspergillus* species^53^. Consequently, it has been suggested that fungal metallo-beta-lactamases may be involved in the degradation of toxic secondary metabolites produced by plant hosts^10^. In this case, this may suggest that *S. sclerotiorum* is upregulating a metallo-beta-lactam-associated gene for the purpose of detoxifying a lactone produced by *B. napus*. Though these *S. sclerotiorum*-plant detoxification interactions have not been characterised, the several differentially expressed domains associated with secondary metabolite modification hint at the possibility of secondary metabolite degradation as a means of interspecific adaptation in *S. sclerotiorum*.

Thee major facilitator superfamily-domain genes were differentially expressed by *S. sclerotiorum* in a host-specific fashion, namely sscle_02g012440 (LFC = 3.9, p_adj_ = 0.004), sscle_05g040320 (LFC = 5.3, p_adj_ = 0.001) and sscle_05g047210 (LFC = 3.2, p_adj_ = 0.004) (Table 3). MFS proteins are involved in the active transport of compounds across cell membranes, and are present in both eukaryotes and prokaryotes^54^. Some MFS proteins are known to export toxic xenobiotic compounds and have been implicated in the resistance of organisms to antibiotics, fungicides and phytotoxins^55,56^. One possibility is that the differentially expressed MFS genes are involved in the detoxification of host-specific phytotoxins. Relatively few membrane transporters are known to be involved in the detoxification of phytotoxins such as phytoalexins, and most of these transporters are ATP-binding cassette ABC transporters^11^. One exception is the MFS-encoding gene *MgAtr5*, which is thought to be involved in the efflux of the phytotoxins resorcinol and resveratrol in *Mycosphaerella graminicola* during infection of wheat^12^. The detoxification role played by *MgAtr5* suggests that MFS-based efflux of phytotoxins could be possible in *S. sclerotiorum*.

This study investigated host-specific gene expression in the fungal necrotroph *S. sclerotiorum* between the host species *B. napus* and *L. angustifolius*. A total of 628 genes were upregulated *in planta*, including a number of secreted CAZymes, putative effectors and secondary metabolite-encoding genes. Of arguably greater interest were the smaller set of 53 genes that were differentially expressed between the two host species. Many of these genes had potential roles in the detoxification of plant-derived secondary metabolites.

Fungal detoxification of phytotoxins has been previously investigated in relation to chemical control of fungal pathogens. New fungicide chemistries could aid the plant’s natural defence responses by inhibiting fungal detoxification-related enzymes, preventing the metabolism of phytotoxins. Further research into detoxification pathways in fungal pathogens could provide additional targets for similar fungicides, potentially improving the variety of fungicides available for controlling pathogens such as *S. sclerotiorum*.

## Materials and Methods

### Biological materials

*B. napus* cv. ‘Cobbler’ and *L. angustifolius* cv. ‘Tanjil’ plants were grown from seed in a plant growth chamber with a 16 hour photoperiod and 16 °C/22 °C temperature cycle. Relative humidity was maintained at 60% and the daytime light intensity was 200 µmol/m^2^/s. *S. sclerotiorum* cultures (isolate CU11.19) were prepared from dry sclerotia on potato-dextrose agar (PDA)^57^. After two days of growth, mycelial plugs were taken from the actively growing edges of the PDA cultures and subcultured onto minimal glucose medium.

For RNA sequencing and qPCR experiment 1, plants were inoculated at 5 weeks post-sowing with 5 mm minimal media agar plugs taken from the actively growing edges of the *S. sclerotiorum* cultures. The plugs were bound to the stem with Parafilm to conserve moisture. *The in vitro* control consisted of *S. sclerotiorum* cultures cultivated in 50 mL of potato-dextrose broth. These *in vitro* cultures were inoculated with 5 mm minimal media agar plugs. For qPCR experiment 2, detached leaves of *B. napus* and *L. angustifolius* plants were inoculated with mycelial matts grown in minimal medium as per Seifbarghi *et al*. (2017)^13^.

The plant sections, mycelial matts and *in vitro* cultures were harvested at 3 days post-inoculation (DPI). Stem sections were cut from the plants at the outer extent of visible tissue necrosis. The plant samples were immediately snap frozen in liquid nitrogen, and stored at −80 °C. The *in vitro* cultures were drained on sterile filter paper and washed twice with MilliQ water, before being blotted dry on sterile filter paper. Subsequently, the *in vitro* samples were snap-frozen in liquid nitrogen and storedat −80 °C.

### RNA extraction and sequencing

RNA was extracted from the samples using a modified TRIzol method (Supplementary Materials) (Invitrogen Corp., Carlsbad, CA, USA). DNA was degraded by the addition of Turbo DNase (Invitrogen Corp., Carlsbad, CA, USA). The absence of genomic DNA was verified via PCR, while RNA yield and quality were assessed via Qubit and gel electrophoresis. The samples were then snap-frozen in liquid nitrogen and stored at −80 °C. RNA sequencing was conducted by Novogene (Wan Chai, Hong Kong, China) using the Illumina (San Diego, CA, USA) HiSeq platform, using 150 bp PE reads and an insert size of 250–300 bp. Raw reads are deposited in the GenBank Sequence Read Archive under accession number PRJNA516496.

### Quality control and adapter removal

Raw Illumina reads were first inspected using the FastQC tool (V. 0.11.8)^58^. Trimmomatic 0.38 was used for quality and adapter trimming^25^. Leading and trailing bases were set at 3, with a sliding window of 4:15 and a minimum length of 36 bp.

### Read assignment and mapping

Trimmed reads from the *in planta* samples were then assigned to either *S. sclerotiorum, B. napus* or *L. angustifolius* using the BBSplit tool (version 38.12)^59^. Reads were assigned to the genome for *S. sclerotiorum* strain 1980 genome (bioproject PRJNA348385, assembly ASM185786v1)^38^ and either the *L. angustifolius* cv. Tanjil (bioproject PRJNA356456, assembly GCA_001865875)^60^ or *B. napus* (bioproject PRJNA293435, assembly GCA_000686985.2)^61^ genomes depending on the sample. The *in vitro* samples were filtered with BBsplit using only the *S. sclerotiorum* genome. The “ambig2” option was set as “toss”, which excluded ambiguous reads. Only the reads that mapped to the *S. sclerotiorum* genome were used for further analysis.

*S. sclerotiorum* reads were aligned to the *S. sclerotiorum* genome assembly using HiSat2 (v2.1.0)^62^. The resulting.sam files were converted to the.bam format using SAMtools v1.9^63^. Aligned reads were assembled using Stringtie v1.3.4, guided by reference gene annotations^38,64^. Gene counts were calculated from the Stringtie assemblies using the PrepDE.py script provided within the Stringtie package^64^.

### Differential expression analysis and quality assessment

Differential expression analysis was conducted in order to determine which genes were significantly differentially expressed between the two hosts, and the *in vitro* sample. The Limma (v3.38.1) package in R was used for this analysis because it has the capacity to compare more than two treatments in a single stage of analysis^26,27^. During the initial screening of the gene expression results, genes with counts-per-million (cpm) values of less than 1 in more than 3 samples were removed, because they were unlikely to be significantly differentially expressed. The remaining read counts were normalised using the “TMM” method.

The differential expression profiles of the samples were visualised using the plotMDS() function from Limma and the heatmap.2() function from gplots^65^. According to these plots, one *B. napus* replicate (C2) and possibly one *L. angustifolius* replicate (L3) appeared to be have a very distinct expression profile from the other replicates. To correct for the influence of these outliers, the voomWithQualityWeights() function was used to weight replicates based on their similarity. Pairwise comparisons were made between all three experimental treatments (*L. angustifolius in planta, B. napus in planta* and *in vitro*). All comparisons were made at the α = 0.05 significance level after false discovery rate (FDR) correction, and only log-fold changes (LFCs) greater than 2 were considered biologically significant.

### Pathogenicity factors and DbCan2 secreted CAZyme prediction

CAZymes, secondary metabolites and proteinaceous effectors are known pathogenicity factors in necrotrophic pathogens such as *S. sclerotiorum*. DbCan2 (v7) was used to predict putative CAZymes from the *S. sclerotiorum* genome^66^. The *S. sclerotiorum* strain 1980 protein sequences were used as the input for CAZyme prediction (GCA_001857865)^38^. The putative CAZYmes predicted by DbCan2 were filtered before use in further analysis. Only CAZymes predicted by at least two of the three algorithms employed by DbCan2 were retained, in order to limit the number of spurious CAZyme predictions. To focus on secreted CAZymes, the predicted CAZymes were restricted to those with positive SignalP results. Putative effectors were obtained from Derbyshire *et al*. and Amselem *et al*.^4,38^. Secondary metabolite genes were identified using AntiSMASH.

### Quantitative polymerase chain reaction

The 15 genes in Supplementary Table 2 were analysed using quantitative PCR (qPCR) as a complementary approach to RNA sequencing for detection of differential expression between samples. We performed two experiments, experiment 1 consisted of samples generated in exactly the same way as for the RNA sequencing experiment. For this experiment, there were three replicates for *in vitro* and *B. napus* derived samples and two for *L. angustifolius* derived samples. All samples were independent of those used for RNA sequencing, so they represent further replication of this analysis. Experiment 2 was performed using methods adapted from Seifbarghi *et al*. (2017)^13^. In this experiment, mycelial matts of *S. sclerotiorum* were first grown on minimal medium inoculated with a PDA plug. The matts were transferred to detached leaves of *B. napus* or *L. angustifolius* and removed for RNA extraction at 3 DPI. Experiment 2 was replicated four times for each condition.

The RNA extracted from these samples was converted to cDNA using the Roche first strand cDNA synthesis kit for RT-PCR (AMV). The cDNA samples were then diluted 1/20 before qPCR. The qPCR analysis was performed using the Bio-Rad iTaq Universal SYBR Green Supermix according to the manufacturer’s instructions. The primers used are detailed in Supplementary Table 3. The thermocycler settings were 95 °C for 2 min, then 95 °C for 15 sec, 60 °C for 30 sec and 72 °C for 15 sec, repeated 40 times, followed by 72 °C for 2 min. Data were analysed using the ∆Ct method, relative to the *S. sclerotiorum β-tubulin* gene. For statistical comparisons, we used Welch’s t-test on log(2^−Δ*Ct*^) values when comparing only two samples. For comparing three samples, we used Analysis of Variance on log(2^−Δ*Ct*^) values.

## Supplementary information

Supplementary meterials

Supplementary tables 1

Supplementry tables 2

Supplementry tables 3

## Data Availability

The trimmed reads have been deposited in GenBank as short read archives under accession number PRJNA516496.
