# Supplementary material for: The host generalist phytopathogenic fungus Sclerotinia sclerotiorum differentially expresses multiple metabolic enzymes on two different plant hosts
Source: Sci Rep. 2019 Dec 27;9:19966. doi: 10.1038/s41598-019-56396-w (PMC6934579; doi:10.1038/s41598-019-56396-w)
Supplement: Supplementary file 1 — Supplementary meterials [file 41598_2019_56396_MOESM1_ESM.docx]

**The host generalist phytopathogenic fungus *Sclerotinia sclerotiorum* differentially expresses multiple metabolic enzymes on two different plant hosts**

Jefferson Allan, Roshan Regmi, Matthew Denton-Giles, Lars Kamphuis and Mark Derbyshire

**Supplementary materials**

RNA extraction methodology

Samples were first ground to a fine powder in an RNase-free mortar and pestle in liquid nitrogen. Aliquots of approximately 100mg were transferred to 2mL Eppendorf tubes. To each sample, 1mL of cold TRIzol was added before being briefly vortexed (Invitrogen Corp., Carlsbad, CA, USA). The samples were then incubated at room temperature for 5 minutes and centrifuged for 10 minutes at 12 000 x g. The supernatant was transferred to a fresh 1.5mL Eppendorf tube, to which 200uL of chloroform was added. The samples were shaken by hand and incubated for 10 minutes at room temperature, before being centrifuged for 10 minutes at 12 000 x g. The separated upper phase was transferred to another 1.5mL Eppendorf tube. The RNA was precipitated by the addition of 250 uL of isopropanol and 250 uL of a saline solution containing 0.8M sodium citrate and 1.2M NaCl. The samples were incubated for 5 minutes at room temperature. The pellet was washed with 70% ethanol and resuspended in 50uL of RNAse-free water. In order to prevent RNA degradation, RNase inhibitor (Applied Biosystems, Waltham, Massachusetts, USA) was added to the samples.
